# Silibinin, a natural flavonoid, induces autophagy via ROS-dependent mitochondrial dysfunction and loss of ATP involving BNIP3 in human MCF7 breast cancer cells

**DOI:** 10.3892/or.2015.3915

**Published:** 2015-04-17

**Authors:** KAI JIANG, WEI WANG, XIN JIN, ZHAOYANG WANG, ZHIWEI JI, GUANMIN MENG

**Affiliations:** 1 Department of Clinical Laboratory, Tongde Hospital of Zhejiang Province, Hangzhou, Zhejiang 310012, P.R. China; 2 Department of Ophthalmology, Xinhua Hospital, Shanghai Jiaotong University School of Medicine, Shanghai 200092, P.R. China; 3 School of Electronics and Information Engineering, Tongji University, Shanghai 201804, P.R. China

**Keywords:** mitochondrial membrane potential, reactive oxygen species, silibinin, autophagy, ATP, BNIP3

## Abstract

Silibinin, derived from the milk thistle plant (*Silybum marianum*), has anticancer and chemopreventive properties. Silibinin has been reported to inhibit the growth of various types of cancer cells. However, the mechanisms by which silibinin exerts an anticancer effect are poorly defined. The present study aimed to investigate whether silibinin-induced cell death might be attributed to autophagy and the underlying mechanisms in human MCF7 breast cancer cells. Our results showed that silibinin-induced cell death was greatly abrogated by two specific autophagy inhibitors, 3-methyladenine (3-MA) and bafilomycin-A1 (Baf-A1). In addition, silibinin triggered the conversion of light chain 3 (LC3)-I to LC3-II, promoted the upregulation of Atg12-Atg5 formation, increased Beclin-1 expression, and decreased the Bcl-2 level. Moreover, we noted elevated reactive oxygen species (ROS) generation, concomitant with the dissipation of mitochondrial transmembrane potential (ΔΨm) and a drastic decline in ATP levels following silibinin treatment, which were effectively prevented by the antioxidants, N-acetylcysteine and ascorbic acid. Silibinin stimulated the expression of Bcl-2 adenovirus E1B 19-kDa-interacting protein 3 (BNIP3), a pro-death Bcl-2 family member, and silencing of BNIP3 greatly inhibited silibinin-induced cell death, decreased ROS production, and sustained ΔΨm and ATP levels. Taken together, these findings revealed that silibinin induced autophagic cell death through ROS-dependent mitochondrial dysfunction and ATP depletion involving BNIP3 in MCF7 cells.

## Introduction

Breast cancer is one of the most common cancer among women aged between 50 and 70. About one million new cases of breast cancers are diagnosed each year worldwide (1). The best lines of defense, radiation therapy and chemotherapy, are unsatisfactory due to the untoward side effects on healthy cells and the problem of drug resistance. Therefore, the development of new therapeutic drugs for breast cancer is clinically important.

Apoptotic (type Ι) and autophagic cell death (type II) are two common forms of programmed cell death (PCD) (2). Autophagy is a degradative process that is characterized by the sequestration of cytoplasmic components in double-membrane vesicles which fuse with lysosomes to form autolysosomes (3). Autophagy plays an important role in cellular homeostasis by acting as a housekeeper to clear damaged organelles such as broken mitochondria, to remove misfolded proteins and to eliminate intracellular pathogens (4). Aside from its basic role in the turnover of proteins and organelles, autophagy also shows multiple physiological and pathophysiological functions in cell differentiation, immune defense and cell death (5). Autophagy has been implicated to play a role in cancer, cardiomyopathy and neurodegenerative diseases (6,7).

Bcl-2 adenovirus E1B 19-kDa-interacting protein 3 (BNIP3), a member of the ‘BH3-only’ subfamily of pro-apoptotic Bcl-2 family proteins which induces cell death, has a key role in the pathogenesis of many diseases, such as cancer (8). BNIP3 is primarily localized to the mitochondria with the N-terminus oriented into the cytoplasm and the C-terminus inside the mitochondria, and has been documented to be the key regulator of mitochondrial permeability transition pore (mPTP) and mitochondrial dysfunction (9). Previous research has shown that overexpression of BNIP3 induces cell death accompanied by rapid and strong mitochondrial dysfunction caused by the alteration of mitochondrial transmembrane potential (ΔΨm) and the opening of mPTP (10,11), which could lead to the production of reactive oxygen species (ROS). Recently, BNIP3 is known as a potent inducer of autophagy in many different cell types (9). As BNIP3 may represent a potential therapeutic target in cancer, it is important to understand how BNIP3 regulates mitochondrial function and autophagy.

Silibinin is a type of flavonoid which is the major bioactive component of silymarin isolated from milk thistle (*Silybum marianum*). It has been used as a traditional medicine specifically as a hepatoprotective drug in Europe and Asia and shows obvious clinical curative effects for jaundice, hepatitis and gallbladder disease (12). Recently, many studies have shown that silibinin has anticancer activities in various types of cancer cells, such as colon, prostate, skin and glioma cancer cells. In these studies, silibinin caused cell death through inhibition of proliferation, activation of the MAPK pathway and induction of inflammation and apoptosis (13,14). However, the mechanisms underlying the antitumor effect of silibinin have not been clearly elucidated. Thus, the present study was designed to elucidate the effect of silibinin on the human MCF7 breast cancer cell line and to determine its underlying mechanisms. Here, we provide evidence that autophagy plays an important role in silibinin-induced cell death. Furthermore, we showed that autophagic cell death induced by silibinin was accompanied by ROS-dependent alteration of ΔΨm and loss of ATP production that involved BNIP3.

## Materials and methods

### Reagents

Silibinin, 3-(4,5-dimethylthiazol-2-yl)-2,5-diphenyltetrazolium bromide (MTT), N-acetylcysteine (NAC), ascorbic acid (AA), 3-methyladenine (3-MA), bafilomycin-A1 (Baf-A1), mouse anti-human BNIP3 and rabbit anti-human light chain 3 (LC3) were all obtained from Sigma-Aldrich. Antibodies for rabbit anti-human Bcl-2, β-actin, rabbit anti-human Atg12 and rabbit anti-human Beclin-1 were all purchased from Cell Signaling Technology (Beverly, MA, USA). 5,5′,6,6′-Tetrachloro-1,1′,3,3′-tetraethylbenzimidazol ylcarbocyanine iodide (JC-1) and dihydroethidium (DHE) were obtained from Molecular Probes. BNIP3 siRNA was purchased from RiboBio Co. (guangdong, China).

### Cell culture

MCF7 cells were cultured in RPMI-1640 media supplemented with 10% fetal calf serum, 100 u/ml penicillin, and 100 *μ*g/ml streptomycin. Cells were incubated at 37°C in a humidified atmosphere of 5% CO_2_ and 95% air. Serum-starved cells were cultured as above without 10% fetal calf serum for 24 h.

### Measurement of cell viability

Cell growth was measured using the MTT assay. Cells were seeded in a 96-multiwell plate at a concentration of 5×10^3^ cells/well. After overnight incubation, the medium was replaced with fresh medium containing silibinin at various concentrations and incubation was carried out for 24 or 48 h. MTT (0.5 mg/ml) was added during the last 4 h of incubation. In each well, the absorbance was measured at 570 nm. For the trypan blue assay, cells were seeded in a 6-multiwell plate at a concentration of 5×10^5^ cells/well in RPMI-1640 media with/without 10% fetal calf serum. After 24 h, 100 *μ*M of silibinin was added and incubation was carried out for 2, 6, 12 or 24 h. Cells were harvested and stained with 0.4% trypan blue solution for 10 min. Non-viable cells were stained blue, and the ratio of blue cells to total cells was recognized as the cell death rate.

### Measurement of ROS

Cells were seeded in a 6-multiwell plate at a concentration of 5×10^5^ cells/well. After overnight incubation, the medium was replaced with a fresh medium containing silibinin at various concentrations and incubation was carried out for 24 h. Next, the cells were harvested and incubation was carried out with 5 *μ*M DHE in PBS for 20 min at 37°C. Then the cells were washed with PBS twice before FACS analysis.

### Measurement of ΔΨm

Cells were seeded in a 6-multiwell plate at a concentration of 5×10^5^ cells/well. After overnight incubation, the medium was replaced with fresh medium containing silibinin at various concentrations and incubation was carried out for 24 h. Next, the cells were harvested and incubated with 5 *μ*M JC-1 in PBS for 20 min at 37°C. Then the cells were washed with PBS twice before FACS analysis.

### ATP measurement

Cells were seeded in a 6-multiwell plate at a concentration of 5×10^5^ cells/well. After overnight incubation, the medium was replaced with fresh medium containing silib-inin at various concentrations and incubation was carried out for 24 h. Next, the cells were harvested and lysed in 1% NP-40 and when the ATP in the lysates and luciferin/luciferase enzyme complex combined, a reaction which produces light occurs. The relative light units were detected by the DTX 880 Multimode Detector (Beckman Coulter).

### RNA interference

The siRNA sequences were: BNIP3-siRNA sense, 5′-CACGAGCGUCAUGAAGAAAUU-3′ starting at nucleotide 439 from the Aug start codon of human BNIP3 coding sequence (GenBank™ accession no. MM 004052) and BNIP3-siRNA antisense, 5′-UUUCUUCAUGACGCUCGUGUU-3′. siRNA (100 nM) was transfected into MCF7 cells using Lipofectamine™ RNAiMax (Invitrogen, Carlsbad, CA, USA) according to the manufacturer’s instructions for ~48 h.

### Quantitative real-time fluorescence polymerase chain reaction (qRT-PCR)

Total RNA was isolated using TRIzol reagent (Invitrogen) from MCF7 cells following treatment with 100 *μ*M silibinin or other reagents. cDNA was synthesized using ReverTra Ace^®^ qPCR RT kit (Toyobo, Japan) according to the manufacturer’s instructions. qRT-PCR reactions were run on an Applied Biosystems 7500 Real-Time PCR system machine. Gene expression levels in all samples were examined using SYBR-Green (Takara, Japan) according to the manufacturer’s instructions. The sequences were as follows: BNIP3 sense, 5′-GTTCCAGCCTCGGTTTCT-3′ and antisense, 5′-AGCCCTGTTGGTATCTTGTG-3′; GAPDH sense, 5′-TGCCAAATATGATGACATCAAGAA-3′ and antisense, 5′-GGAGTGGGTGTCGCTGTTG-3′. After correcting the levels of expression of each mRNA to GAPDH, the relative levels of transcripts in the MCF7 cells were calculated using the 2^−ΔΔCt^ method (15).

### Western blot analysis

Cells were extracted in lysis buffer (Cell Signaling Technology) and the lysates were separated on a 12.5% SDS-PAGE using the SDS-PAGE system as described (16). Proteins were then transferred to a PVDF membrane (Millipore, Billerica, MA, USA) in transfer buffer. After blocking with 5% non-fat dried milk for 2 h, the membrane was incubated with the primary antibodies overnight at 4°C. Then the immunoreactive bands were visualized by enhanced chemiluminescence using HRP-conjugated secondary antibodies and blots were developed by an enhanced chemiluminescence detection system (Amersham-Pharmacia Biotech).

### Statistical analysis

Data (mean ± SEM) were statistically analyzed using one-way analysis of variance (ANOVA) followed by Dunnett’s test. P<0.05 was considered to indicate a statistically significant result.

## Results

### Silibinin induces autophagic cell death

**Figure 1:**
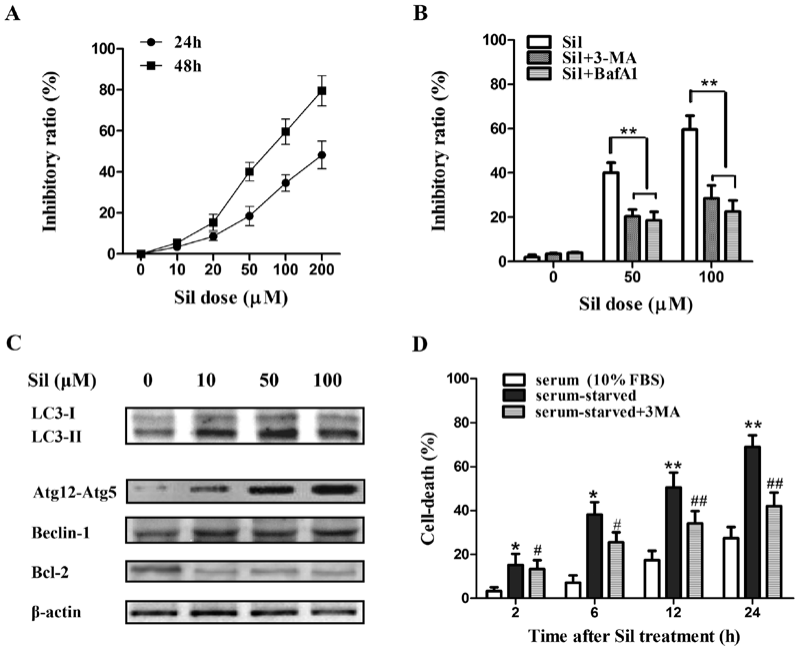
Effects of silibinin on cell viability and autophagy. (A) MCF7 cells were treated with various concentrations of silibinin (0–200 *μ*M) for 24 and 48 h. Cell viability was determined by MTT assay. (B) Effect of pretreatment with autophagy inhibitors 3-MA or Baf-A1 on autophagic death by silibinin in the MCF7 cells. ^**^P<0.01 vs. the silibinin group (n=3). (C) Cells were exposed to various concentrations of silibinin (0–100 *μ*M) for 24 h, and expression levels of autophagy-related proteins were detected by western blot analysis. (D) Cell death induced by silibinin (100 *μ*M) under serum-starved condition or serum-sufficient condition. Cell death was determined by trypan blue assay. ^*^P<0.05, ^**^P<0.01 vs. the serum-sufficient (10% FBS) group; ^#^P<0.01, ^##^P<0.01 vs. the serum-starved group (n=4). 3-MA, 3-methyladenine; Baf-A1, bafilomycin-A1.

First, we assessed the efficacy of silibinin against the cell viability of MCF7 human breast cancer cells. The cells were exposed to various concentrations of silibinin for 24 and 48 h, and the cell viability was measured by MTT assay. Silibinin caused a marked increase in cell death in a time- and concentration-dependent manner (Fig. 1A). Then two autophagy inhibitors, 3-MA (which blocks the initial stages of autophagy by inhibiting class III PI3 kinase) and Baf-A1 (which blocks autophagosome-lysosome fusion by inhibiting the vacuolar H^+^-ATPase), were further used to investigate whether silibinin-induced cell death is attributed to autophagy. MCF7 cells were treated with 5 mM 3-MA or 50 nM Baf-A1 for 2 h prior to 24 h of silibinin treatment (100 *μ*M). 3-MA and Baf-A1 pretreatment both greatly abrogated silibinin-induced cell death (Fig. 1B), indicating that autophagy contributed to the cell death in the MCF7 cells by silibinin. Next, to further confirm the induction of autophagy by silibinin, a set of autophagy-related factors including LC3-I and LC3-II, Atg12-Atg5 formation, and Beclin-1 in the MCF7 cells after treatment with silibinin (0–100 *μ*M) for 24 h were investigated by western blot analysis. Notably, the conversion of LC3-I to LC3-II (LC3-II/LC3-I ratio), an established indicator of autophagy, was greatly enhanced by silibinin (Fig. 1C). The formation of LC3-II is known to depend on the Atg12-Atg5 conjugate, and Atg12-Atg5 formation was significantly elevated (Fig. 1C), confirming the activated autophagy by silibinin. Moreover, the expression of Beclin-1, which plays a vital role in the regulation of early stages of autophagosome formation, was increased although the increase was not as marked as the level of LC3-II or Atg12-Atg5 formation. Recently, Bcl-2 was reported to act as an anti-autophagy protein via its inhibitory interaction with Beclin-1 (17). As expected, an obvious decrease in the level of Bcl-2 was observed (Fig. 1C). Notably, MCF7 cells treated with silibinin (100 *μ*M) under serum-starved conditions underwent an immediate increase in cell death at 2 h, which occurred earlier than that under serum-sufficient (10% FBS) conditions, and the autophagy inhibitor 3-MA partially inhibited cell death by silibilin under this circumstance (Fig. 1D), suggesting that the MCF7 cells were more sensitive to silibinin-induced autophagic cell death under the starvation condition.

### Silibinin causes ROS generation and mitochondrial dysfunction

**Figure 2:**
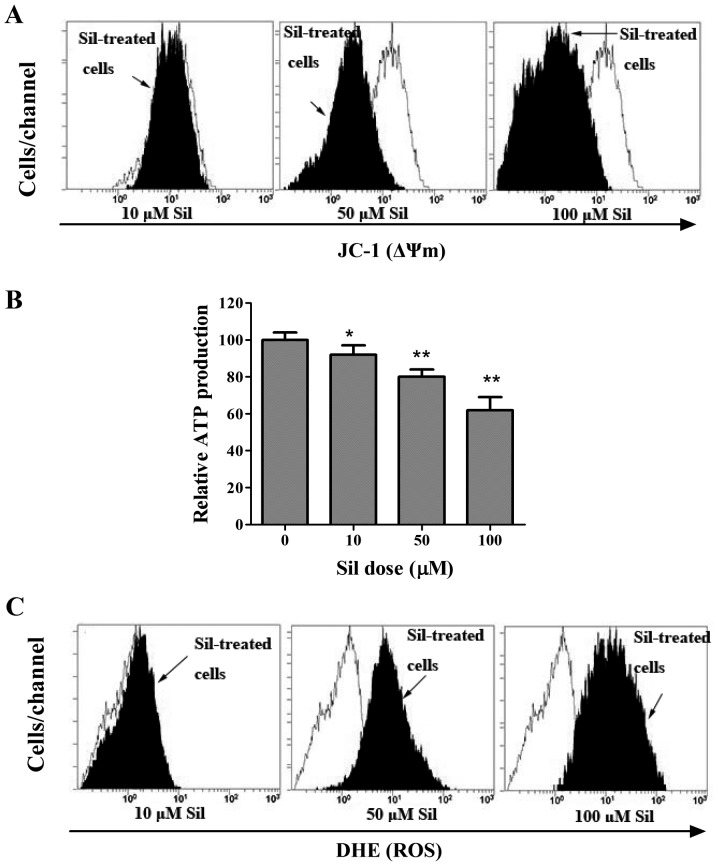
Effects of silibinin on mitochondrial transmembrane potential (∆Ψm), ATP production and ROS generation. MCF7 cells were treated with various concentrations of silibinin, after which the control cells (white profiles) and silibinin-treated cells (black profiles) were labeled either with the ΔΨm-sensitive dye JC-1 to detect ∆Ψm (A), or the probe DHE to detect ROS generation (C) by FACS analysis. (B) MCF-7 cells were treated as indicated and harvested, and the total cellular ATP content was measured by the luminometric ATP assay. ^*^P<0.05, ^**^P<0.01 vs. the control (n=3). DHE, dihydroethidium; ROS, reactive oxygen species.

To elucidate the mechanism of the induction of autophagic cell death by silibinin, we next investigated the mitochondrial activities during silibinin treatment. ΔΨm, as an important parameter of mitochondrial function, is often employed as an indicator of cellular viability. Thus, we adopted a potential-dependent fluorescent carbocyanine and lipophilic dye 5,5′,6,6′-tetrachloro-1,1′,3,3′-tetraethylbenzimidazolylcarbocy-anine iodide (JC-1), which accumulates in the mitochondria, to indicate the dissipation of ΔΨm. As shown in Fig. 2A, in the control cells, the bright red fluorescence generated by JC-1 was indicative of high membrane potential, whereas silibinin caused a dose-dependent reduction in ΔΨm, as indicated by a shift in the fluorescence peak to the left. Since ΔΨm is known to be essential for various functions including the production of ATP via oxidative phosphorylation (18), we sought to determine whether loss of ΔΨm caused changes in ATP content. As expected, cellular ATP levels progressively declined in the silibinin-treated MCF7 cells (Fig. 2B), suggesting mitochondrial dysfunction. The generation of ROS is known to be induced by the opening of the mPTP (19). Thereby, the fluorogenic probe, DHE, was used to assess the effect of silibinin on ROS generation. Compared to the control cells, the cells stained with DHE showed a progressively enhanced ROS level by exposure to increasing concentrations of silibinin (Fig. 2C).

### Antioxidants prevent silibinin-triggered ROS generation as well as autophagic cell death, mitochondrial dysfunction and ATP depletion

**Figure 3:**
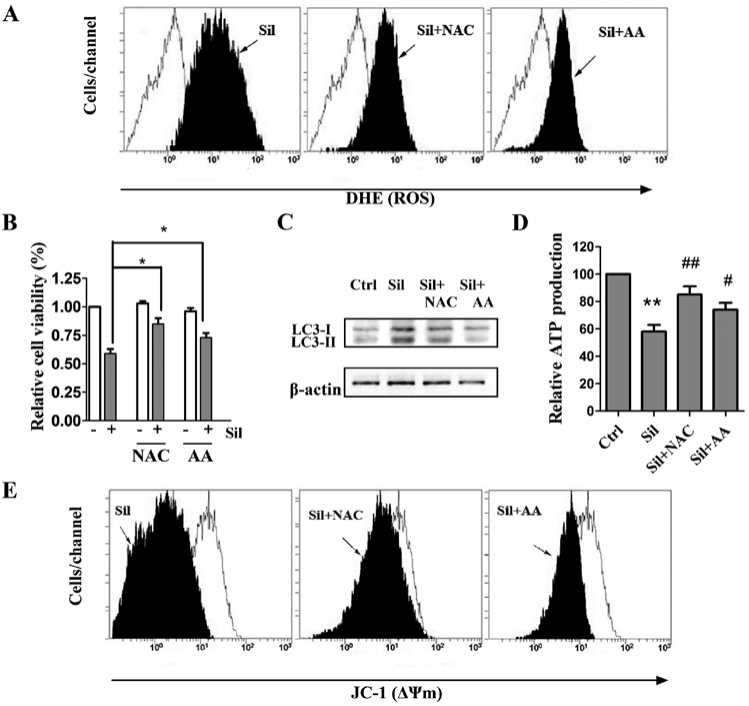
Effect of ROS on silibinin-stimulated autophagic cell death as well as mitochondrial dysfunction and ATP depletion. MCF7 cells were pretreated with ROS scavengers NAC (5 mM) or AA (500 *μ*M) for 2 h, and stimulated with 100 *μ*M silibinin (Sil) in the presence or absence of the drugs for 24 h. (A) Inhibition of ROS production by the antioxidants. (B) Involvement of ROS in silibinin-induced cell death by MTT assay. ^*^P<0.01 vs. the silibinin group (n=3). (C) Effect of ROS on autophagy-related LC3-I and LC3-II expression. The bands are representative of at least 3 separate experiments. (D) Inhibition of ROS production recovered ATP production as detected by the luminometric assay. ^**^P<0.01 vs. the control; ^#^P<0.05, ^##^P<0.01 vs. the silibinin group (n=3). (E) Effect of ROS on silibinin-induced mitochondrial dysfunction. White profiles, control cells; black profiles, cells treated with silibinin together with the antioxidants. ROS, reactive oxygen species; NAC, N-acetylcysteine; AA, ascorbic acid; LC3, light chain 3.

The pivotal role of ROS in mediating silibinin-induced cell death has previously been revealed (20), and it has also been reported that ROS could regulate autophagy in various cell models (21). Therefore, we investigated whether ROS scavenging could inhibit silibinin-promoted autophagy by adopting two antioxidants, NAC and AA, known as strong scavengers of ROS. Cells were pretreated with 5 mM NAC and 500 *μ*M AA for 2 h and stimulated with silibinin (100 *μ*M) for another 24 h. We observed that the antioxidants exerted little effect on the intracellular ROS levels (data not shown). Notably, pretreatment with both NAC and AA greatly inhibited the silibinin-stimulated ROS generation (Fig. 3A). After verifying the inhibition of ROS production by the antioxidants, the involvement of ROS in silibinin-induced autophagic cell death and mitochondrial dysfunction was then investigated. As shown in Fig. 3B and C, pre-incubation with NAC or AA effectively prevented not only the loss of cell viability but also the elevated autophagic LC3-II/LC3-I ratio, indicating that autophagic cell death was ROS-dependent. Notably, pretreatment of the antioxidants effectively recovered ATP levels and ΔΨm during silibinin treatment (Fig. 3D and E). These results indicate that the ROS generation may be a cause of silibinin-triggered autophagic cell death and mitochondrial dysfunction as well as ATP depletion.

### BNIP3 mediates silibinin-induced cell death

**Figure 4:**
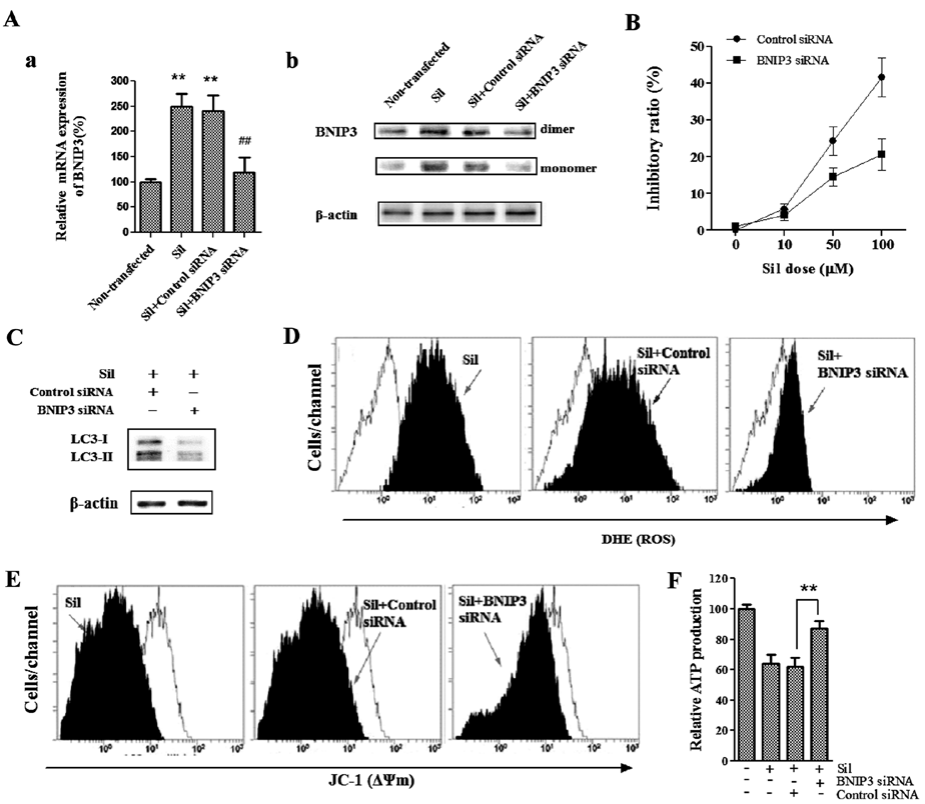
Silibinin induces BNIP3-mediated cell death. MCF7 cells were transfected with 100 nM siRNA targeted to BNIP3 or control siRNA for 48 h, and further incubation with 100 *μ*M silibinin was carried out for an additional 24 h. (A) mRNA (a), and protein expression (b) of BNIP3 was detected by qRT-PCR and western blotting using mRNA and cell lysates. ^**^P<0.01 vs. the control; ^##^P<0.01 vs. the silibinin group (n=3). Silencing of BNIP3 inhibited silibinin-induced cell death (B), conversion of LC3-I to LC3-II (C), ROS production (D), as well as loss of ∆Ψm (E) and ATP depletion (F). ^**^P<0.01 vs. silibinin + control siRNA group (n=3). BNIP3, Bcl-2 adenovirus E1B 19-kDa-interacting protein 3; LC3, light chain 3; ROS, reactive oxygen species; ∆Ψm, mitochondrial transmembrane potential.

To determine whether BNIP3, a pro-death protein and a member of the BH3-only Bcl-2 family, is required for silibinin-induced cell death, we used BNIP3-specific siRNA to knock down BNIP3 and then analyzed the effects of loss of BNIP3 on silibinin-enhanced autophagic cell death as well as ROS production and mitochondrial dysfunction. As shown in Fig. 4A, silibinin upregulated BNIP3 protein and transcript levels, whereas BNIP3 siRNA blocked BNIP3 upregulation induced by silibinin. We found that the cells pretreated with BNIP3 siRNA were more resistant to silibinin-induced cell death as compared to those pretreated with the control siRNA (Fig. 4B). Furthermore, silencing of BNIP3 significantly reduced the silibinin-enhanced autophagy-related LC3-II/LC3-I ratio (Fig. 4C), suggesting that BNIP3 was essential for the silibinin-induced autophagic cell death noted in the MCF7 cells. Since silencing of BNIP3 effectively reversed the cell death and autophagy induced by silibinin, we next investigated the involvement of BNIP3 in silibinin-induced ROS production, as well as loss of ΔΨm and ATP production. Notably, BNIP3 siRNA greatly abrogated the ability of silibinin to induce an increase in ROS production (Fig. 4D), as well as loss of ΔΨm (Fig. 4E) and decrease in ATP production (Fig. 4F). Together these data provide evidence that BNIP3 plays a critical role in the silibinin-induced autophagic cell death, as well as ROS generation, mitochondrial dysfunction and defective ATP production.

## Discussion

Silibinin, a popular dietary supplement isolated from milk thistle seed extracts, has shown considerable efficacy in treating various types of cancer. Recently, it has been reported that silibinin effectively induced apoptosis, type Ι of PCD, in a human breast carcinoma cell line (22), suggesting its potential as a therapeutic agent against breast cancer. However, the mechanisms underlying the antitumor effect of silibinin are poorly defined. Notably, our present study demonstrated that silibinin induced MCF7 human breast cancer cells to undergo autophagic cell death (PCD type II), as well as ROS-dependent disruption of ΔΨm and loss of ATP production through a mechanism involving BNIP3.

It is well-known that excessive levels of autophagy can lead to cytoplasmic vacuolation resulting in cell death (23). In the present study, silibinin-induced autophagic cell death was demonstrated, as 3-MA and Baf-A1, two autophagy inhibitors acting at different stages of autophagy, effectively abrogated silibinin-induced cell death. In addition, we investigated a set of autophagy hallmarks, including the conversion of LC3-I to LC3-II, Atg12-Atg5 formation, and Beclin-1 to further confirm the induction of autophagy. The Atg12-Atg5 conjugate is known as one of the key stages for the autophago-somes and it acts as an E3-like enzyme for lipidation of Atg8 family proteins and their association to vesicle membranes in autophagy (24). Microtubule-associated protein 1-light chain 3 (LC3) is the mammalian homolog of the yeast protein Atg8. When autophagy is activated, LC3-I is activated by Atg7 and modified into the active form LC3-II (membrane-bound) which is the component of autophagosomes (25). Consistent with these data, we found that both the level of LC3-II and the LC3-II/LC3-I ratio which closely correlate with the number of autophagosomes, as well as Atg12-Atg5 formation were increased after exposure to silibinin. Our finding that elevated expression of Beclin-1 which was accompanied by a decreased level of Bcl-2 is significant, since Beclin-1 participates in the regulation of the early stages of autophagosome formation (26), and Bcl-2 is reported to inhibit autophagy by interacting with Beclin-1 and can repress mTOR to activate autophagy via preventing Ca^2+^ release from ER (27). It has been reported that the PCD pathways I and II may both be induced by the same stimuli and/or in the same cell types (28). Therefore, it is possible that silibinin-induced autophagy was accompanied by apoptosis, and the interplay between apoptosis and autophagy occurred, which warrants further investigation.

Besides its role in removing mitochondria damaged by oxidative stress, autophagy also plays a role in the catabolism of oxidized proteins (29). Previous data indicate that ROS plays an essential role in the activation of autophagy (30). It was demonstrated that ROS can induce autophagy through several distinct mechanisms involving Atg4-Atg8/LC3, Beclin-1, PI3K-Akt-mTOR, catalase, and the mitochondrial electron transport chain, which leads to both cell-survival and cell-death responses and could be selective toward cancer cells (31,32). In the present study, silibinin treatment caused a concentration-dependent increase in intracellular ROS. Furthermore, the antioxidants NAC and AA prevented not only ROS production but also silibinin-induced autophagy, indicating that ROS production seems to be a cause of silibinin-induced autophagic cell death. Similar to what we observed, several pieces of evidence indicate that silibinin induces autophagic death through the ROS pathway in other cancer cell types, such as human fibrosarcoma HT1080 (33) and HeLa cells (34).

Notably, our findings demonstrated that the ATP levels were significantly reduced by silibinin in the MCF7 cells. ATP is a switch to cell death and mitochondrial dysfunction induces changes in cellular energy metabolism in cancer cells (35). It has been reported that high-polarized (high ΔΨm) mitochondria help the maintenance of sufficient ATP levels (36). Similar to previous findings, we noted that the loss of ΔΨm was accompanied by a marked decline in the cytoplasmic ATP content, indicating destruction of mitochondrial function by silibinin. In addition to their critical role in ATP synthesis, mitochondria are also the major source of ROS in most cell types (35). Mitochondrial dysfunction is usually characterized by the opening of mPTP, a non-specific pore in the inner mitochondrial membrane. The opening of mPTP results in massive swelling of the inner membrane and subsequent rupture of the outer mitochondrial membrane, causing release of mitochondrial components into the cytoplasm including ROS (37). In this regard, we initially speculated that the dissipation of ΔΨm caused by silibinin might contribute to the generation of ROS. Surprisingly, we found that inhibition of ROS production by antioxidants effectively regenerated ΔΨm and maintained ATP levels, implying that ROS are responsible for silibinin-stimulated mitochondrial dysfunction and subsequent ATP depletion. This finding is supported by the facts that excessive ROS trigger the opening of mitochondrial channels, and in turn, leads to the simultaneous collapse of the ΔΨm and a transient increase in ROS generation. This mitochondrion-to-mitochondrion ROS signaling constitutes a positive feedback mechanism involving the recently described process named ROS-induced ROS-release (RIRR) which may release an ROS burst finally leading to cell death (38).

BNIP3 is a mitochondrial BH3-only protein that contributes to cell death through activation of the mitochondrial pathway of apoptosis (11). Although it belongs to the Bcl-2 family, its pro-cell death activity is distinct from other family members. BNIP3 is also known to induce mitochondrial autophagy (9). In the present study, our results showed that silencing of BNIP3 significantly reduced the silibinin-enhanced autophagy-related LC3-II/LC3-I ratio, implying an essential role of BNIP3 in silibinin-induced autophagic cell death in MCF7 cells. Notably, BNIP3 siRNA sustained ΔΨm and ATP levels during autophagy, and prevented ROS production in the silibinin-treated MCF7 cells. Similar to what we observed, Ghavami *et al* showed that overexpression of ∆TM-BNIP3, a dominant-negative BNIP3 mutant which prevents wild-type (wt) BNIP3 from targeting the mitochondria and antagonizes wt BNIP3-induced effects, not only reversed cell death and autophagy, but also reduced ROS production and mitochondrial damage in S100A8/A9-treated cells including MCF7 (39).

**Figure 5:**
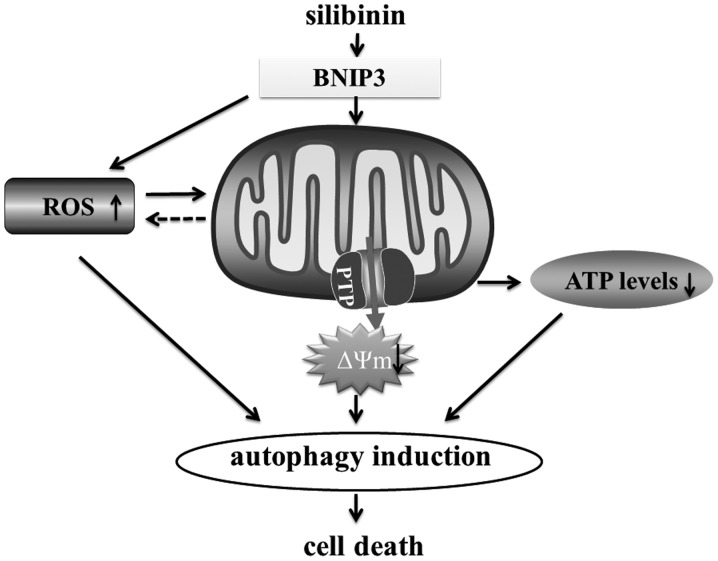
Schematic diagram of the potential molecular mechanism of MCF7 cell death by silibinin.

In conclusion, the present study shed new light on the mechanisms involved in silibinin-triggered cell death. The present study suggests that silibinin-induced autophagic cell death involves an increase in ROS production, subsequently followed by mitochondrial dysfunction and ATP depletion. Finally, we suggest that BNIP3 is the critical factor that mediates the ROS-dependent mitochondrial death pathway (Fig. 5).
